# MiR-99b-5p expression and response to tyrosine kinase inhibitor treatment in clear cell renal cell carcinoma patients

**DOI:** 10.18632/oncotarget.12618

**Published:** 2016-10-12

**Authors:** Magdalena Lukamowicz-Rajska, Christiane Mittmann, Michael Prummer, Qing Zhong, Jens Bedke, Jörg Hennenlotter, Arnulf Stenzl, Axel Mischo, Svenja Bihr, Manuela Schmidinger, Ursula Vogl, Iris Blume, Christoph Karlo, Peter Schraml, Holger Moch

**Affiliations:** ^1^ Institute of Surgical Pathology, University Hospital Zürich, Zurich, Switzerland; ^2^ NEXUS Personalized Health Technologies, ETH Zürich, Zürich, Switzerland; ^3^ Department of Urology, University Tübingen, Tübingen, Germany; ^4^ Oncology Department, University Hospital Zürich, Zürich, Switzerland; ^5^ Department of Internal Medicine I, Division of Oncology & Comprehensive Cancer Center Vienna, Medical University of Vienna, Vienna, Austria; ^6^ Institute for diagnostic and interventional Radiology, University Hospital Zurich, Zürich, Switzerland

**Keywords:** renal cancer, ccRCC, miR, sunitinib, treatment response, microRNA, tyrosine kinase inhibitors

## Abstract

A number of treatments targeting VEGF or mTOR pathways have been approved for metastatic clear cell Renal Cell Carcinoma (ccRCC), but the majority of patients show disease progression after first line therapy with a very low rate of complete or long-term responders. It has been shown that miRs may play a role in prediction of treatment response in various cancer types. The aim of our study was to identify a miR signature predictive for RCC patients' response to antiangiogenic tyrosine kinase inhibitor (TKI) treatment in the first line therapy. Sequencing of 40 paired normal/tumor formalin fixed and paraffin embedded ccRCC tissues revealed separate clustering via unsupervised dendrograms. With supervised analysis, the strongest differential expression was obtained with miR-99b-5p, which was significantly lower in patients with short progression free survival (<8 months) and TKI non-responders (progressive disease patients according to RECIST) (p<0.0001, each). Validation using RTqPCR and a second patient cohort compiled from three different hospitals (n=65) showed higher expression of miR-99b-5p in complete responders, but this trend did not reach statistical significance. It is concluded that low miR-99b-5p expression analyzed with sequencing methodology may correlate with tumor progression in TKI-treated ccRCC patients.

## INTRODUCTION

Renal cell carcinoma (RCC) accounts for 2% of all cancers. Up to 30% of patients present with metastases at diagnosis and have a poor prognosis with a 5-year survival rate of approximately 8% [1]. Most RCC are classified as clear cell subtype (ccRCC) that is characterized by frequent mutations of the von Hippel-Lindau (VHL) tumor suppressor gene [2]. In the absence of VHL protein, Hypoxia-inducible factor 1α (HIF1α) and Hypoxia-inducible factor 2α (HIF2α) are stabilized and, as a consequence, modulate the transcription of various HIF target genes, such as Platelet-Derived Growth Factor (PDGF), Vascular Endothelial Growth Factor (VEGF) or mammalian Target Of Rapamycin (mTOR).

A standard treatment procedure in metastatic RCC involves systemic therapy, but the majority of patients show disease progression after first line therapy [3]. Until 2006, there were limited treatment options available and overall survival for patients was only 12 months [4]. Since 2006 novel treatments targeting PDGF and VEGF pathways (Sutent (sunitinib), Nexavar (sorafenib), Avastin (bevacizumab)) as well as mTOR pathway (Everolimus (afinitor) and Temsirolimus (torisel)) have been approved for metastatic RCC treatment [5].

MiRs (miRNAs, microRNAs) belong to a group of short non-coding RNAs that bind to 3′ end of the messenger RNA (mRNA) mediating its translational repression and/or degradation. Therefore, they likely play a significant role in regulation of apoptosis, cell proliferation and differentiation. The “seed sequence” of the miR, a fragment of the miR sequence responsible for mRNA recognition, may bind to one or multiple mRNAs. Moreover, one mRNA may be a target of various miRs. The role of miR in the regulation of renal cancer biology has been intensively investigated [6–15]. Numerous miRs were reported to be significantly deregulated in various RCC subtypes [8–10, 12, 14, 16, 17]. MiR-141 [9, 10, 14, 16, 17] and miR-200c [6, 8–10, 14, 16, 17] were significantly down regulated in ccRCC, and were suggested to bind to the mRNA of VEGF [14, 18], whereas hypoxia related miRs miR-210 [8–10, 12, 14, 16, 17, 19], miR-155 [8–10, 12, 14, 16, 17], and miR-21 [8, 12, 16] were up-regulated in ccRCC.

The identification of predictive biomarkers is of utmost importance to improve the outcome of RCC patients [20]. Literature reports suggest that micro RNAs may play a role in prediction of tumor recurrence [21] and in resistance of RCC to chemotherapy or targeted therapies. Moreover miRs deregulation has been associated with the risk of metastasis after nephrectomy [22] or response to the RCC treatment therapies [11, 23–25]. Nakada et al. [11] correlated increase of miR-210 to chemotherapy resistance using renal cancer cell lines. In contrast to sunitinib sensitive patients, a significant down-regulation of miR-141 was observed in ccRCC patients that did not respond to sunitinib treatment [23]. Twenty eight miRs were differentially expressed in patients with poor response compared to patients with good response [24]. MiRs potentially predictive for sunitinib response were investigated in ccRCC metastases [25] and elevated miR-942 levels were linked to sunitinib resistance. However, no reports attempting to identify markers of sunitinib response tested on non-clinical trial patient cohorts using a comprehensive next generation sequencing approach, enabling analysis of all known miRs within one sample and in a single analysis, exist.

Therefore, the goal of our study was to identify and validate miRs that identify patients resistant/susceptible to tyrosine kinase inhibitor (TKI) treatment by next generation sequencing. MiR candidates selected based on sequencing analysis were further validated using a different assay platform and tissue and serum samples of an independent RCC patient cohort.

## RESULTS

### miR expression and progression free survival (PFS)/RECIST response classification correlation

**Figure 1:**
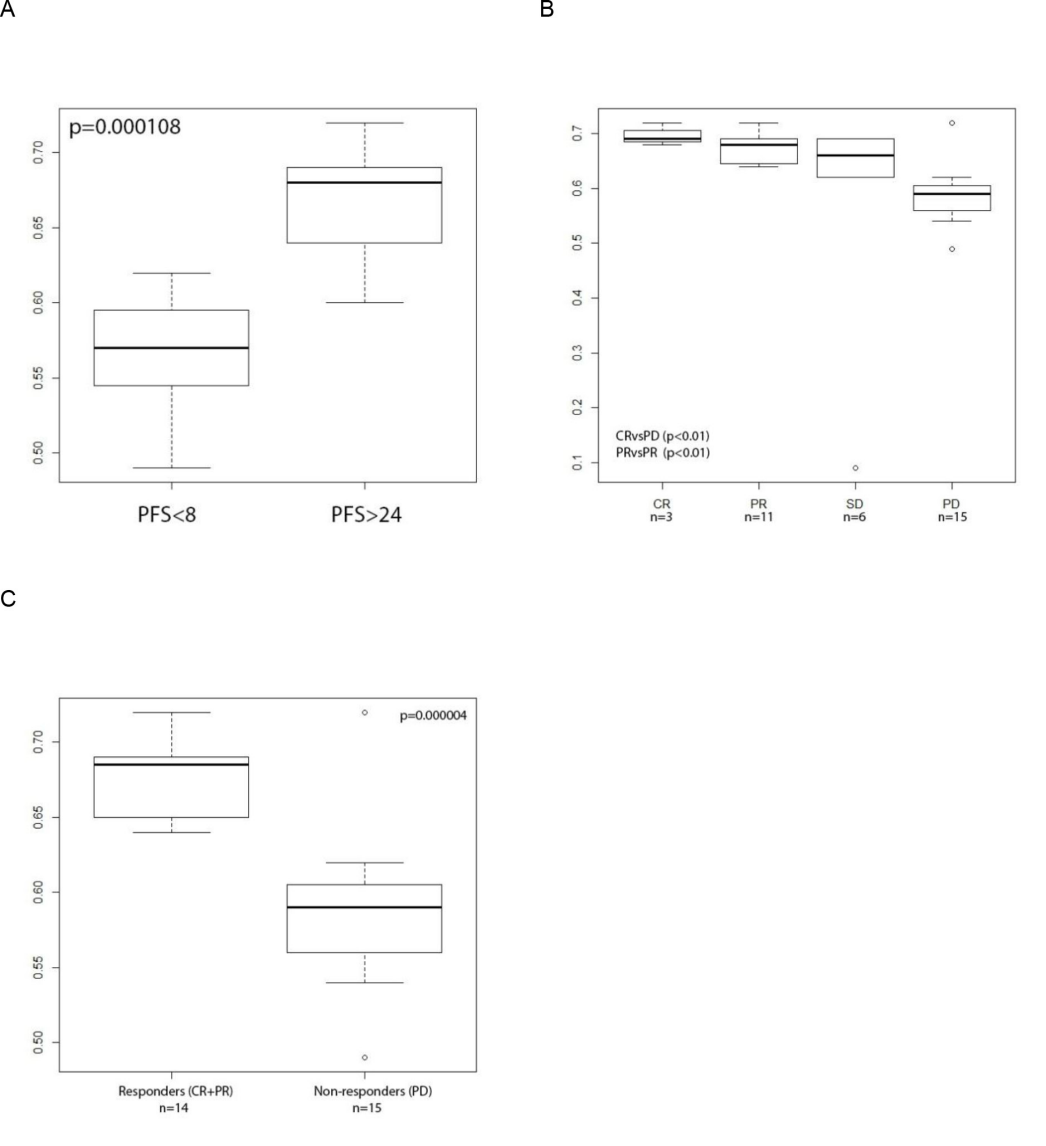
MiR-99b-5p expression in tumor tissue, measured with sequencing platform normalized with SOR **A**: box plots of two groups (PFS<8 months; n=8 and PFS>24 months; n=9) showing the most extreme PFS values. On the X axis two groups of progression free survival (PFS) values expressed in months are presented, on the Y axis the miR expression values are scaled. Independent on the normalization (in the figure data of SOR normalization method are presented) method low miR-99b-5p expression successfully defined early progression patients (PFS<8 months) **B**: box plot of the RECIST scoring groups and **C**: responders vs non-responders. As the “n” the number of patients per group is presented, p value indicate the statistical significant difference between the groups. Independent on the normalization method (in the figure data of SOR normalization method are presented) low miR-99b-5p expression successfully defined patients with progressive disease. PFS – progression free survival, PR- progressive disease, SD- stable disease, PR- partial response, CR- complete response.

In order to exclude any bias originating from the data normalization we applied 3 normalization techniques and selected the candidate miR that correlated with treatment response independent of the normalization method. Sequencing data of were normalized via sum of reads (SOR), quantile normalization (Q) and DESeq2 normalization (DESeq2). All miRs with expression of five reads or lower (SOR and Q) or on average 1 read (DESeq2) were not considered adequate for further analysis (Supplementary Table 1). PFS data were correlated with each miR expression using Spearman's rank correlation. MiRs that showed a statistically significant correlation (p< 0.05) in all three normalization approaches are presented in Table 1. The strongest positive correlation in all normalization methods was shown for miR-99b-5p. For the extreme phenotype selection [26–28] approach (patients with PFS lower than 8 months vs. patients with PFS higher than 24 months) the expression levels in the two groups were compared using Student's t-test. The most significant difference was observed for miR-99b-5p (e.g.: SOR: p=1.1*10^−4^, Figure 1A). A higher miR expression was observed for longer PFS, whereas early progressive patients exhibited low miR-99b-5p expression.

**Table 1:** Spearman's rank correlation of miR expression obtained with sequencing platform and progression free survival values that were statistically significant (*p*<0.05) with all three normalization methods: normalization via sum of reads (SOR), quantile normalization (Q) and DESeq2-normalization (DESeq2)

	Spearmans rank correlation					
	SOR		Q		DESeq2	
	correlation	*p* value	correlation	*p* value	correlation	*p* value
hsa-miR-301a-3p	−0.6907	0.0008	−0.5824	0.0143	−0.5413	0.0291
hsa-miR-423-5p	−0.6332	0.0016	−0.6230	0.0057	−0.5783	0.0169
hsa-miR-501-3p	0.4968	0.0187	0.5547	0.0193	0.5349	0.0318
hsa-miR-100-5p	0.5614	0.0082	0.5648	0.0181	0.5325	0.0318
hsa-miR-99b-5p	0.6465	0.0016	0.6836	0.0011	0.6431	0.0062

All presented miRs passed the filter of 5 reads (SOR and Q) or on average 1 read (DESseq2) for all samples. Values below 0 indicate negative correlation (the higher PFS the lower miR expression), whereas correlation >0 prove that miR expression increases with PFS values (positive correlation).

miR-99b-5p was further correlated with RECIST response to the treatment (Figure 1B-1C).: a statistically significant difference was noted if patients with CR (n=3) or PR (n=11) were compared with patients with PD (n=15) (e.g.: SOR: CR vs PD p=5.16*10^−4^, PR vs PD p=1.2*10^−5^). Since the CR group consisted only of three patients and PR and CR defined the objective response, responders were combined and statistically compared with the non-responder group (PD). For miR-99b-5p a strong significance was obtained (e.g.: SOR: p=4*10^−6^) (Figure 1C). Higher miR expression correlated with tumor shrinkage after TKI treatment (responders).

### MiR's expression-based prediction of progression free survival and response classification

Next we used classification random forest analysis to investigate whether miR expression predicts the treatment outcome for individual patients. A clear separation of the two RECIST groups, PD and PR, was obtained. Classification of the whole data set of PD and PR patients (n=25), using all 541 informative miRs achieved an out-of-bag accuracy of 96%. One PR patient was erroneously predicted to be non-responsive. In order to define miRs most important for outcome prediction we performed the variable selection procedure. We found 17 miRs to be important for prediction of the test set based on the sudden drop in importance (Supplementary Table 2). A new random forest using only preselected important miRs was built to predict the test set. The out-of-bag accuracy exceeded 90%, limited only by the number of tested patients. All five PD and all five PR patients were classified correctly. Upon more in-depth exploration it turned out that two miRs were sufficient to achieve the same accuracy: miR-99b-5p and miR-100-5p.

**Figure 2:**
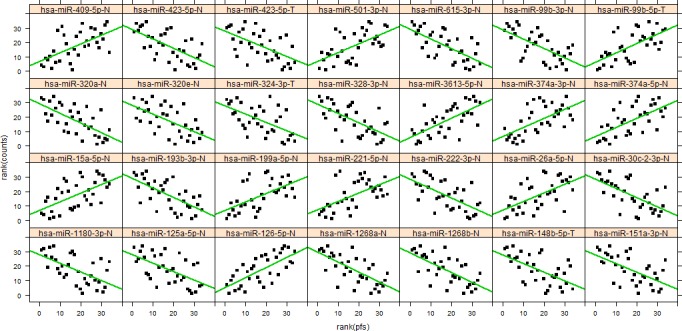
Rank correlation between miR expression and progression free survival (PFS) Shown are the 28 miRs with the lowest p-values with the green line illustrating the fitted Spearman's linear rank correlation.

We also investigated the association of the level of specific miR expression with progression free survival (PFS) by testing for a linear correlation. To avoid any influence of possible outliers, we decided to use Spearman's rank correlation (Figure 2). After post hoc multiple testing corrections (False Discovery Rates<0.05) we obtained a list of 98 miRs with significant rank correlation with PFS (list of the top 20 is shown in Supplementary Table 3).

A summary of the RECIST differential expression analysis and the PFS correlation analysis is shown in Supplementary Figure 1. Fifteen of 29 differentially expressed miRs in the RECIST analysis were also found as hits in the PFS correlation analysis: miR-99a-5p, miR-99b-5p, miR-100-5p, miR-145-3p, miR-151a-3p, miR-199a-5p, miR-199b-5p, miR-328-3p, miR-374a-5p,miR-409-5p, miR-423-3p, miR-423-5p, miR-501-3p, miR-1271-5p, miR-3613-5p.

**Figure 3:**
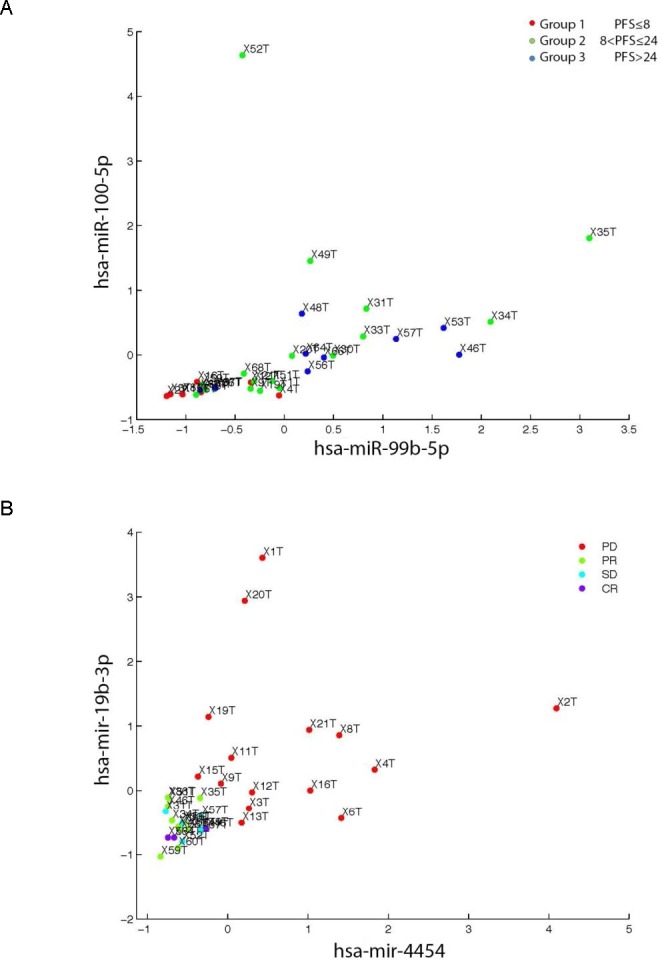
Random forest analysis of miR data using the sequencing approach **A**: The two top candidate miRs (hsa-miR-100-5p, hsa-miR99b-5p) associating with PFS were evaluated. Group 1 (PFS≤8 months; n=8) Group 2 (8<PFS≤24; n=18) and 24<PFS (n=9) as a group 3. E.g.: X1T indicate patient 1 tumor sample and analogously for the other patients labels. Combination of the two top miRs selected based on the random forest analysis showed grouping of patients from group 1 (PFS<8 months) from group 2 and 3 (PFS ≥ 8 months). Such a marker/markers may potentially improve selection of the ccRCC patients for the TKI treatment. **B**: two top candidate miRs (hsa-miR-19b-3p, hsa-miR-4454) evaluated using the RECIST labeling. PD patients presented as red dot, SD as blue dot, PR as green dot and CR as violet dot. E.g.: X1T indicate patient 1 tumor sample and analogously for the other patients labels. Presented top targets successfully showed distinct grouping of PD patients after TKI treatment was applied. ccRCC - clear cell renal cell carcinoma, PFS - progression free survival, PD- progressive disease, SD- stable disease, PR- partial response, CR- complete response, TKI - tyrosine kinase inhibitor.

In the following step a classification random forest analysis with differently defined labels was performed. Two data labels were applied: PFS (group 1: PFS<8, group 2: 8≤PFS≤24, group 3; PFS>24 months) and RECIST (PD, SD, PR, CR). Using a-priori defined patient clinical data allowed us to formulate a list of 10 top miR candidates that defined different patient groups.

the following 10 miRs were identified in PFS analysis: miR-99b-5p, miR-100-5p, miR-21-3p, miR-221-3p, miR-4508, miR-93-5p, miR-454-3p, miR-126-3p, miR-423-3p, miR-660-5p listed from the strongest candidate (with the plotted top two miRs presented in Figure 3A).

the following 10 miRs were identified for RECIST: miR-4454, miR-19b-3p, miR-423-3p, miR-151a-3p, miR-532-5p, miR-183-5p, miR-423-5p, miR-328-3p, miR-221-3p, miR-99b-5p listed from the strongest candidate (with the top two miRs plotted in Figure 3B).

MiR-99b-5p was the only miR among the top 10 hits, which was identified independently of the criteria chosen (PFS or RECIST classification). These data strengthen the results obtained with unsupervised clustering or the random forest regression analysis in regards to miR-99b-5p to be the top candidate.

### Differential miR expression as predictor of TKI response

**Figure 4:**
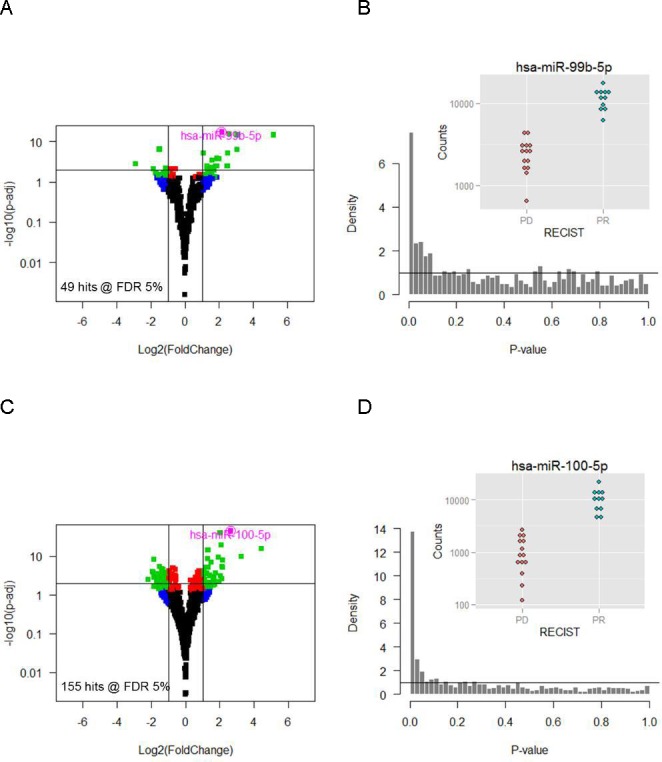
Differential expression between PR and PD patients in tumor (A, B) and normal tissue (C, D) of sequencing cohort Volcano plots (**A**, **C**) relating p-values to fold-changes show significant (PFS<8 months) and strongly (> 2-fold) DE miRs in green. The corresponding p-value distributions (**B**, **D**) are highly regular confirming the validity of the analysis. Inset: raw counts of the top scorer miR-99b-5p (B) and miR-100-5p (D).PR- partial response, PD- progressive disease.

Differential miR expression between partial responders (PR) and patients with progressive disease (PD) were compared separately in normal and tumor tissue to explore the possibility of using miR expression to predict TKI treatment response. Independent filtering excluded 977 miRs with mean count <2 reads from the analysis (67 % out of 1455 with non-zero total read count) in tumor and/or normal tissue. Univariate testing produced 49 hits at False Discovery Rates <0.05 after multiple testing correction (Figure 4A). The most significant candidate was miR-99b-5p, which was upregulated approximately 4-fold in partial responders (Figure 4B). The resulting p-value distribution was normal indicating a good correspondence between data and model requirements.

Likewise, independent filtering excluded 755 miRs with a mean count <0.7 from the analysis (57% out of 1325 with nonzero total read count) in tumor and/or normal tissue. We found 155 hits with False Discovery Rates <0.05 and miR-100-5p was the most significant candidate with a close to 10-fold overexpression in PR patients (Figure 4C and 4D).

### Validation of the sequencing results by RTqPCR

As additional control, the results of 10 of 40 sequenced tissues of ccRCC patients were evaluated by RTqPCR using 5 control miRs. The RTqPCR results correlated well with sequencing data (R^2^_min_=0.80 to R^2^_max_=0.99) for all but two patients (Supplementary Table 4). MiR-155 and miR-210 showed a greater up regulation if analyzed with RTqPCR (294- and 214733-fold respectively) than with sequencing (22- and 14-fold respectively) in patient 8. In contrast to the expected up regulation of miR-155, based on sequencing results (22-fold) a minor downregulation was observed by RTqPCR (0.9-fold) in patient 12.

**Figure 5:**
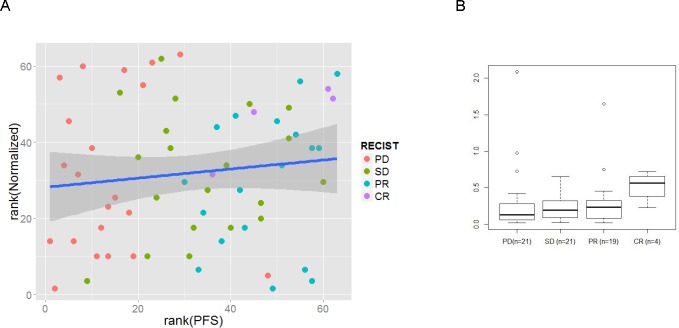
RTqPCR results for the miR-99b-5p expression analysis (n=61) Mean value of three control RNAs (RNU44, RNU 48 and U6 snRNA) has been used as reference for normalization of miRs expression levels. **A**: The Spearman's rank correlation analysis of progression free survival (PFS, X=axis) and the miR-99b-5p expression (Y axis) resulted in 0.21 (p=0.10), that indicate low statisticaly insignificant correlation, in contrast to high correlation observed for miR sequencing data. **B**: the analysis of RECIST classes and its correlation with miR-99b-5p expression. No statistically significant correlation observed (two tailed student's t-test).

MiR-99b-5p proved to be our top candidate identified by sequencing. Lower miR-99b-5p expression was observed in patients with short PFS and in PD patients compared to patients with long PFS and responders. Therefore, miR-99b-5p was selected for further validation using a Taqman RT-qPCR platform in 65 patients (already sequenced and non-sequenced patients) from Zurich (n=16), Vienna (n=33) and Tübingen (n=16). MiR-99b-5p was detected in 61 of 65 patients. MiR-99b-5p was below the detection level in four patients. Spearman's rank correlation analysis revealed no correlation between PFS and miR-99b-5p expression level (Figure 5A). The group with early progression (PFS<8 months, n=29) was further analyzed against the group with late progression (PFS>24 months, n=19), but there was no different miR-99b-5p expression level (two-tailed student's t-test, p=0.67). Also, no statistically significant difference of miR expression difference was found if different RECIST groups were analyzed (Figure 5B), however, higher miR-99b-5p expression was observed for CR patients if compared to all other groups.

### Serum analysis

We further investigated miR-99b-5p expression in nine healthy donors to determine presence of miR-99b-5p in patient serum. The normalization assays have been evaluated experimentally based on the literature data (see supplements “Serum normalization method assessment”). MiR-99b-5p was detected in the serum of healthy donors with the Ct value below 38 cycles for all analyzed samples (Supplementary Figure 2).

**Figure 6:**
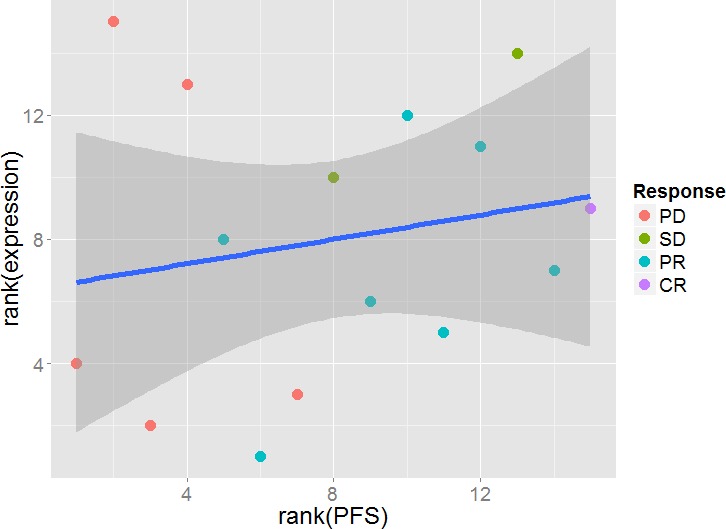
RTqPCR results for the miR-99b-5p expression analysis in 15 ccRCC patients' serum samples No significant correlation was noted if miR expression (Y axis) was correlated with PFS values (X axis).

Our analysis included additional 15 serum samples of ccRCC patients from Tübingen. We found no correlation of PFS and circulating systemic miR-99b-5p levels (Figure 6) in tumors, suggesting that miR-99b-5p expression levels in patient serum is not useful to identify responders from non-responders to TKI.

### Survival analysis of the candidate hsa-miR-99b-5p

**Figure 7:**
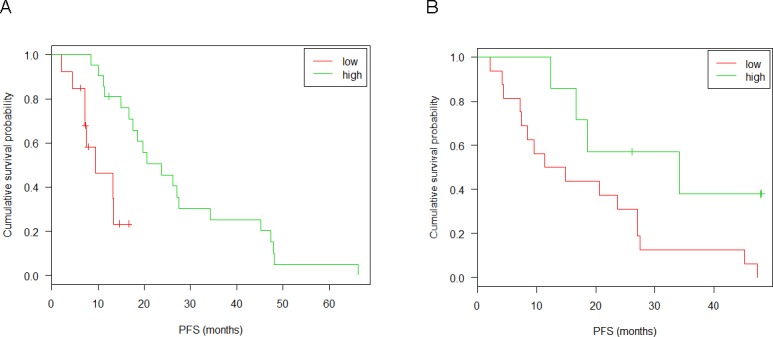
Kaplan-Meyer plots and survival analysis for miR-99b-5p of tumor tissue in the discovery group Discovery group: patients from UHZ and Vienna that were sequenced. (**A**) miRNAseq: split point 0.12; sample size 13(low), 21(high); p-value 0.002; 95% confidence interval [0.1; 0.5]. (**B**) RTqPCR: split point 0.42; sample size 15(low), 7(high); p-value 0.002; 95% confidence interval [0; 0.7]. Censored observations are marked by crosses. Results of the likelyhood ratio test are given in the inset. Patients were distributed into low and high expressor groups according to a log-rank test.

**Figure 8:**
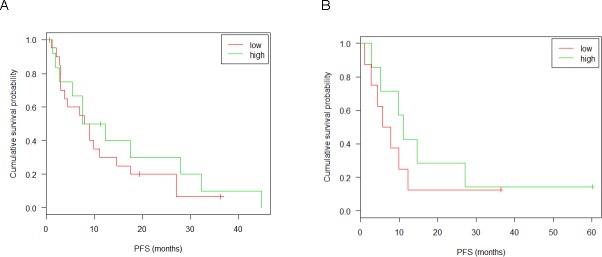
Kaplan-Meyer plots and survival analysis for miR-99b-5p in the confirmation group Discovery group: patients from UHZ, Vienna or Tübingen that were not sequenced. (**A**) RTqPCR of tumor tissue: split point 0.18; sample size 20(low), 12(high); p-value 0.457; 95% confidence interval [0.3; 1.6]. (**B**) RTqPCR of serum from 15 patients of the Tübingen cohort: split point 0.11; sample size 8(low), 7(high); p-value 0.401; 95% confidence interval [0.2; 1.9]. Censored observations are marked by crosses. Results of the likelyhood ratio test are given in the inset. Patents were distributed into low and high expressor groups according to a log-rank test.

In order to confirm the result of the described correlation test, the association of hsa-miR-99b-5p expression in tumor tissue with PFS was further evaluated using the Cox proportional hazard model properly taking censoring into account. To this end, the patient population was optimally split into a high (21 patients) and a low expressing group (13 patients) according to the log-rank test. This yielded a significant association of the risk of progression with miRNA expression in the UZH and Vienna cohort, as illustrated in Figure 7A. This observation could be confirmed by RTqPCR of the same tumor samples (Figure 7B). Neither tumor samples of the confirmation group (patients from Zürich, Vienna and Tübingen cohorts) (Figure 8A) nor serum samples (Figure 8B) from patients of the Tübingen cohort did show an expression-dependent risk association.

## DISCUSSION

In this study, we aimed to identify predictive miRs in tissue samples from ccRCC patients who were treated for their initial metastatic disease with sunitinib in a non-clinical trial approach. We observed that high miR-99b-5p expression levels, measured with next generation sequencing, are associated with longer PFS (p<0.05, significant association of the PFS with miRNA expression – survival analysis). MiR-99b-5p was also the best candidate in predicting response to sunitinib treatment, defined according to RECIST criteria. A statistically significant difference was seen if CR or PR as well as responders (grouped CR and PR patients) were compared with PD patients (p<0.05 for all sets). Notably, sequencing data analysis of RCC patients treated with sunitinib showed association of PFS and/or RECIST response with additional members of the miR-99 family. MiR-99a-5p expression correlated with PFS in two out of three (Q and DESeq2) normalization method used. Moreover, stratification of miR expression and PFS correlation with supervised clustering of the sequencing results (random forest), listed miR-100-5p, together with miR-99b-5p, as one of the two top miR candidates. These results suggest a regulatory role of the miR-99 family in the tumor cell response to sunitinib and/or TKI. Interestingly, all mature miR-99 family members are highly conserved among 58 different species and have an identical seed region sequence [29], indicating highly universal targets among the family members. MiR-99b has been already implicated to predict treatment response in other cancer types including prostate [30] and pancreatic [31] cancer. MiR-99b is involved in tyrosine kinase related signaling pathways [14, 31–34], suggesting a major role in response to systemic TKI therapies. As mRNA of mammalian Target Of Rapamycin (mTOR) is one of the top targets of the miR-99 family [14, 32–34], higher levels of these miRs may down regulate mTOR expression, which in turn could inhibit tumor cell growth.

Several studies have described different methodologic approaches and technical platforms to both, tumor classification of RCC and prediction of RCC treatment response based upon miR signatures. Available study results, however, are rather diverse and hard to compare. Gamez-Pozo et al. [24] analyzed the miR profile of 44 patients diagnosed with RCC describing 28 miRs that were differentially expressed in leukocytes isolated from patients with poor response (progression before 6 months) in comparison to patients with good response (no disease progression until 18 month) to sunitinib treatment. This miR selection may rather be leucocyte- than tissue-specific as we found no correlation between miR profile expression and response prediction in our data set. Another group [25] using RTqPCR and applying an extreme phenotype selection [26–28], which was more restrictive than our approach, subdivided RCC patients according to time to progression (>22 months and <5 months) and defined nine potentially predictive candidates for sunitinib efficacy. MiR-942 was the only candidate, which proved its superior value in prediction of sunitinib efficacy with sensitivity of 92% and specificity of 50%. In our analysis, miR-942 was poorly expressed in ccRCC tissue and did not pass the five reads cut off value. Discrepant data was even obtained with miR-141 that was used as control miR in our study. We confirmed significant downregulation in ccRCC samples (FFPE) compared to adjacent non-tumor tissue that is consistent with literature reports [6, 8–10, 12, 14, 16, 17, 19]. However miR-141 was not associated with PFS or RECIST classification that contrasts results of Berkers and colleagues [23] who analyzed fresh frozen tissue samples from RCC patients by RTqPCR and in situ hybridization. They showed a significant down-regulation of miR-141 in ccRCC patients that did not respond to sunitinib treatment compared to TKI sensitive patients [23].

We used RTqPCR to test whether our results yielded with next generation sequencing could be confirmed with a different technique. Although a higher miR-99b-5p expression was observed for CR patients when compared to other groups (PD, SD or PR), this difference did not reach statistical significance. Survival analysis of RTqPCR yielded a significant association of the PFS with miRNA expression in the same patients as selected for sequencing, providing a technical control, however the same analysis performed on non-sequenced patient samples did not confirm this dependence. This fact could suggest the cohort specific association of miR-99b-5p expression and PFS or response to TKI treatment and not the overall dependence. In addition, given the relatively high Ct values obtained for miR-99b-5p with RTqPCR, it is tempting to speculate that RTqPCR method has a generally lower sensitivity for miR detection than sequencing.

Prediction of TKI treatment response in ccRCC with miR profiling is very complex since one miR may regulate expression of many mRNAs and one mRNA may be the target of multiple miRs. We have identified a large number of miRs potentially associated with treatment response or PFS based on sequencing data. The expression levels of miRs might thus indeed help to predict response to TKI treatment for each individual patient. Additionally, specific miR expression patterns have not only been detected in tissue but also in serum of patients with various diseases [13, 24, 35], suggesting that small non-coding RNAs represent potential biomarkers for disease monitoring. Although we observed detectable levels of miR-99b-5p in the serum of both healthy donors and ccRCC patients, there was no correlation with clinical or pathological data. This suggests that miR-99b-5p secretion is not higher in serum of patients with TKI treatment response or characterized with long PFS.

As patient cohorts derived from three different clinical centers, treatment conditions chosen by the (uro)-oncologists for each patient may vary. It cannot be excluded that miR-based predictive values of RECIST class membership or PFS calculation depend on the composition of the selected patients. In contrast to clinical trials in which well-defined and large patient cohorts are analyzed, it is very difficult to extract appropriate data from retrospective oncology reports by focusing on equally treated patients. The limitation of clinical data assessment shifts the focus of attention to a general problem in translational clinical research, particularly, if the prognostic and predictive value of molecular signatures should be evaluated intensively [36].

In summary, comprehensive miR profiling by means of sequencing in TKI treated ccRCC patients revealed high miR-99b-5p expression levels that correlated with both long PFS and response according to RECIST criteria, however this trend did not reach statistical significance if analyzed with RTqPCR platform. Next generation sequencing platforms may be superior to conventional RTqPCR methods, particularly if low expressed miRs are investigated for clinical applications.

## MATERIALS AND METHODS

### Patients' samples

Formalin-fixed, paraffin-embedded (FFPE) and fresh frozen tumor and adjacent non-tumor tissue was obtained from 90 patients diagnosed with advanced metastatic clear cell RCC. Tissue samples collected from 1998 to 2012 were provided by the Department of Pathology, University Hospital Zurich (Zurich, Switzerland) (n=22), Department of Pathology, University of Vienna (Vienna, Austria) (n=39) and University Department of Urology Tübingen (Tübingen, Germany) (n=29). All patients underwent full or partial nephrectomy as part of their standard treatment prior to any treatment. All patients were treated with antiangiogenic TKIs (sunitinib (n=76), sorafenib (n=7) or pazopanib (n=7)) post nephrectomy procedure at the time metastatic disease was diagnosed. Each patient was classified as complete responder (CR), partial responder (PR), stable disease (SD) or progressive disease (PD) according to the RECIST criteria [37, 38]. Progression free survival (PFS) was defined as the time from treatment initiation to disease progression [37, 38] and overall survival (OS) was defined as the time from the first day of treatment to the date of death or last follow-up. The study was approved by the local ethical committee (Ref. nr, EK: KEK-ZH-Nr 2011-0072/4). A summary of the patients' characteristics is provided in Supplementary Table 5.

Tissue samples of RCC patients were snap frozen in pre-cooled isopentane at −80°C (fresh frozen) or formalin fixed and paraffin embedded (FFPE) directly after surgery and stored at −80°C or at RT, respectively. Peripheral blood aliquots for serum samples were prospectively collected at the nephrectomy day and stored at −80°C. Hematoxylin and eosin (HE) stained sections of fresh frozen and FFPE RCC tissues were classified by one specialized uropathologist according to the 2016 WHO classification [39]. Only ccRCC were selected for this study. Tumor and adjacent non-tumor FFPE tissues were punched [40] to obtain 4-8 tissue cylinders (diameter 0.6 mm) used for the RNA isolation. Frozen material was processed in sections.

### RNA isolation

Total RNA was isolated using Qiagen FFPE miRNeasy Kit (Qiagen, Germany)/miRNeasy Mini Kit (Qiagen, Germany) for the FFPE and frozen tissues, respectively, and Qiagen miRNeasy Serum/Plasma Kit for isolation of total RNA from serum following the manufacturer's protocol [41, 42]. Concentration and purity of the RNA were examined by measuring RNA's optical density using the NanoDrop ND-1000 Spectrophotometer (Thermo Scientific, Wilmington, DE, USA).

### RTqPCR assay

TaqMan RTqPCR assay was performed according to the manufacturer's recommendation [43]. In brief, 5ng of total RNA was reverse transcribed using the miR specific stem loop primers (Life Technologies, USA). Obtained cDNA was amplified using ViiA™ 7 Real-Time PCR System (Life technologies, USA). MiR expression data were analyzed using SDS software (Life Technologies, CA, USA).

Five miRs (miR-200c, miR-141, miR-21, miR-210, miR-155) known to be deregulated in ccRCC [6, 8–10, 12, 14, 16, 17, 19] were used as control to validate the tissue.

### Hybridization based miR Assay

Affimetrix QuantiGene miRNA Assay was applied to analyze 5 pre-selected miR (miR-200c, miR-141, miR-21, miR-210, miR-155) in the “ccRCC tissue testing using control miRs” step. All procedures followed the recommendation of the manufacturer (supplementary figure 3) [44]. In brief, tissue was lysed and incubated with miR specific probe sets. The signal was amplified and detected by luminescence detector.

### Sequencing

In order to optimize the library preparation step samples selected for pilot sequencing studies were divided into two aliquots and proceeded with and without ribosomal depletion step. In the main sequencing step, library preparation was performed without ribosomal depletion step.

Samples without ribosomal depletion step: All sequencing libraries were prepared with TrueSeq Small RNA kit (Illumina, USA) according to the producers' protocol and recommendations [45]. Briefly, 1μg of total RNA was ligated with the 3′ and as follows 5′ adapters, reverse transcribed and amplified. cDNA product was purified in the gel purification step that selects the DNA between 160 and 145 base pairs corresponding to the 22-30 nucleotides RNA fragments. DNA libraries were sequenced using the Illumina sequencer (Illumina, USA).

Samples with ribosomal depletion step: Prior to the standard library preparation protocol, described above, an additional ribosomal depletion step was introduced. For this purpose Ribo-Zero Magnetic Kit (Illumina, USA) was used according to manufacturer's recommendation [46].

### Data analysis

### RTqPCR data analysis

Analysis of the quantitative TaqMan RTqPCR data was performed using the SDS 2.4 software (Applied Biosystems). Mean value of three endogenous control RNAs (RNU44, RNU 48 and U6 snRNA) have been used as reference for normalization of miR's expression levels in tissue [47]. A different set of reference control RNAs was recommended for the serum analysis [47]. RTqPCR results were normalized to miR-191 expression in serum analysis (detailed description of the control miR evaluation in a supplementary paragraph “Serum normalization method assessment”). The relative expression levels of target miRs were determined by the equation 2-ΔCT, in which ΔCT was calculated as follows:

ΔCT=CT miR - CT control

### Sequencing data normalization and analysis

The data obtained with the Illumina sequencer were analyzed with Vmatch system software in order to obtain the number of miR copies [32].

### Normalization via sum of reads (SOR)

The relative expression of the annotated miR was calculated by relating the number of reads of the miR to the sum of total miR reads obtained for the analyzed sample.

### Quintile normalization (Q)

In order to unify the miR expression distributions in statistical properties parameter a standard quintile normalization was applied to the total set of data. The normalization was performed in environment of statistical language R [48]. The method was based upon the concept of a quantile-quantile plot extended to n dimensions. No special allowances were made for outliers.

### DESeq2 normalization

The DESeq2 normalization (DESeq2) normalization of data was performed with R/bioconductor environment [49] using the DESeq2 package [50] following the described standard workflow. Briefly, raw count data were compared between groups by use of negative binomial generalized linear models. For this purpose, a sample specific library size factor, a gene specific dispersion parameter, and a gene-specific log2 fold change was estimated. Along the process, automatic independent filtering of low count data and automatic outlier handling was performed

### Statistical analysis

MiR expression data were statistically evaluated in environment of statistical language R by use of Bioconductor and LIMMA package combined with unsupervised hierarchical clustering [51, 52]. To define the correlation of the miR expression and PFS a Spearman's rank correlation and a p values were calculated.

### MiR's differential expression analysis

Differential expression (DE) of each miR for normal and tumor tissue was analyzed in the R/bioconductor environment [49] using the DESeq2 package [50] following the described standard workflow. Following the DESeq2 normalization unpaired testing was applied except for tumor versus normal DE analysis, where the paired nature of the data was considered.

### Extreme phenotype selection and classification random forest feature selection

In order to define the most reliable miR candidates we used the extreme phenotype selection [26–28] approach, comparing patients with PFS lower than 8 months (n=8) with patients for which PFS>24 months (n=9). The statistical significance of the difference between the analyzed groups was evaluated with a two tailed Student's t-test.

In order to define the potential miR targets dependent on the defined labels (PFS or RECIST criteria) a random forest algorithm was performed. In this application, ensembles with 100 trees were grown. The feature importance was estimated by permuting the values of each feature for every observation in the data set. The extent to which the classification error alters after permutation was measured. The procedure was repeated for each feature and the result was summarized in a vector, in which important features exhibit large increase in mean squared error (MSE). We chose the top ten important features for each set of annotations.

### Prediction of treatment response – classification random forest analysis

Machine-learning-based supervised classification of PR and PD patients was performed using the random forest classification algorithm [53] as implemented in R [54]. The remaining two RECIST classes SD and CR were omitted because they were only sparsely populated. Only miRs with more than 1 count on average over all samples were included to build the prediction model (541 miRs in total). Performance of the random forest classifier is measured using the out-of-bag (OOB) error.

For variable selection, the data set was split into a balanced test set of 5 patients in each of the 2 groups and a training set of the remaining 9 PD and 6 PR patients. Subsequently, the average importance of each miR from 50 random forest classifications of the training set was computed and used as a guide to select those which are repeatedly found among the most important ones. For model validation, a new random forest with only the selected important miRs was built to predict the test set and to evaluate the out-of-bag prediction error.

### Correlation of miR expression with PFS

Association of miR expression with progression free survival (PFS) was tested using Spearman's rank correlation. Multiple comparison correction was performed by detailed analysis of the p-value distribution [55, 56] and a false-discovery cut off of 5% was chosen.

### Survival analysis of the candidate hsa-miR-99b-5p

The association of expression of the candidate miRNA hsa-miR-99b-5p on PFS was confirmed using classical survival analysis. The optimal split of the patient population into a lower and a higher risk group was determined with the help of a log-rank test of a conditional survival tree, as implemented in the function tree of the R package party [57]. Significance of the group difference was determined using the Cox proportional hazard model including a test of the assumption using Schönfeld residuals [58].

## SUPPLEMENTARY RESULTS











## ABBREVIATIONS

TKI: tyrosine kinase inhibitor
RCC: Renal cell carcinoma
ccRCC: clear cell renal cell carcinoma
VHL: von Hippel-Lindau tumor suppressor gene
HIF1α: Hypoxia-inducible factor 1α
HIF2α: Hypoxia-inducible factor 2α
PDGF: Platelet-Derived Growth Factor
VEGF: Vascular Endothelial Growth Factor
mTOR: mammalian Target Of Rapamycin
miR: miRNAs, microRNAs
mRNA: messenger RNA
FFPE: formalin-fixed, paraffin-embedded
PFS: progression free survival
CR: complete responder
PR: partial responder
SD: stable disease
PD: progressive disease
OS: overall survival
HE: hematoxylin and eosin
RTqPCR: reverse transcription quantitative polymerase chain reaction
SOR: normalization via sum of reads
Q: quantile normalization
DESeq2: DESeq2 normalization
R: ribosomal depletion step
DE: differential expression
RBC: red blood cells
