# Supplementary material for: MiR-99b-5p expression and response to tyrosine kinase inhibitor treatment in clear cell renal cell carcinoma patients
Source: Oncotarget. 2016 Oct 12;7(48):78433–47. doi: 10.18632/oncotarget.12618 (PMC5346651; doi:10.18632/oncotarget.12618)
Supplement: Supplementary file 1 [file oncotarget-07-78433-s001.pdf]

MiR-99b-5p expression and response to tyrosine kinase inhibitor treatment in clear cell renal cell carcinoma patients

SUPPLEMENTARY RESULTS

Exploratory sequencing step

Studies aimed at the identification of potential markers of TKI response in ccRCC patients included the sequencing of tumor and adjacent non-tumor tissues from 40 ccRCC patients (20 cases from Zurich cohort, 20 cases from the Vienna cohort). Patients were selected for this step based on the following criteria: i) availability of the PFS parameter (at the date of analysis); ii) reliable RECIST classification to represent each RECIST group: PD, SD, PR, CR; Moreover, miRs' expression was evaluated using the Vmatch software for sequencing data analysis with an application of miR v20 database (results of the Vmatch analysis are presented in supplementary table 6).

In order to define the control miRs' expression sequencing data were normalized with sum of reads (SOR) and relative value of tumor vs. non-tumor tissue was evaluated. For all but two patients, patient 10 and patient 16, the deregulation pattern was as expected (supplementary figure 4). To further validate the results for the aforementioned two patients, RTqPCR was performed. The results obtained with this method for patient 16 were consistent with the sequencing data. The down regulation of miR-155-5p in tumor vs non-tumor adjacent tissue was also observed (0.82-fold) (supplementary figure 5). Therefore the results for patient 16 were regarded as valid. In contrast to the sequencing results, up regulation of miR-21-5p by RTqPCR was observed (12.2-fold) for patient 10. Due to the contradictory results of the RTqPCR and sequencing platform as well as due to low read number patient 10 was excluded from further analysis.

ccRCC tissue testing using control miRs

The general description and sequence of the experimental procedures are presented in Supplementary figure 6. To determine FFPE and frozen tissue comparability, the tumor tissue of one patient was analyzed for the expression levels of five key miRs known to be deregulated in ccRCC [1-9]: miR-21, miR-210, miR-155, miR-141, miR-200c using the RTqPCR platform and hybridization based miR assay. Levels of expression of aforementioned miRs demonstrated a high comparability in our analysis (FFPE vs. frozen tissue analysis performed with RTqPCR and hybridization based miR assay; R^2=0.518 and R^2=0.98 respectively). In both, FFPE and fresh frozen tumor tissue, a significant up-regulation of miR-21, miR-210, and miR-155 and down regulation of miR-141 and miR-200c was observed, if compared to the adjacent non-tumor tissue for both assays (supplementary table 7). A significantly higher amount of tissue samples was required for the hybridization based assay. Six cylinders of FFPE tissue were sufficient to perform at least 200 RTqPCR reactions, whereas six tissue cylinders were needed to complete the analysis of 5 miRs in duplicates with the hybridization based assays. Based on these results, FFPE tissue was used for the subsequent experiments.

Pilot sequencing

Tumor and non-tumor tissues from three patients, classified as PR, SD or PD, were selected to adjust the library preparation protocol to the FFPE samples for the pilot sequencing step. Samples were preceded according to the standard library preparation procedure (-R) [10]. In parallel for the same set of samples an additional ribosomal depletion step (+R) was applied in order to investigate its potential improvement in miR sequencing [11]. 488 miRs and 499 miRs out of 2044 investigated were detected with the –R and the +R version of the protocol, respectively. Since no significant improvement was observed after the ribosomal depletion step incorporation, the –R protocol was selected for the main sequencing step.

Confirmation of all control miRs showed a significant down- and up-regulation for the miR-200c (0.01-0.1), miR-141 (0.03-0.1) miR-21 (2.1-14.15), miR-210 (5.1-75.7) and miR-155 (1.2-21.9) as expected based on literature reports. The control miRs sequencing data were normalized with sum of reads (SOR), and relative value of tumor vs. non-tumor tissue was evaluated.

Unsupervised hierarchical clustering

Unsupervised hierarchical clustering analysis was performed in order to compare the tumor and non-tumor tissue samples and to control sequencing data and tissue separation. This analysis correctly stratified the tumor (T) and non-tumor (N) tissue with a single exception (supplementary figure 7, supplementary figure 8). Sample 53T (tumor tissue, patient 53) was classified as non-tumorous tissue (supplementary figure 7). Indeed, re-evaluation of histology revealed that the tissue was non-tumorous tissue.

Serum normalization method assessment

In order to define most reliable control miR we have evaluated 5 different miR candidates chosen based on the literature communications [12, 13]. MiR-16 is the most often referred reference miR in the serum application [12]. MiR-191-5p used by some authors as a serum reference miR, proved to be the most stable in our data cohort if the sequencing data were analyzed. As an externally added and not expressed by human technical quality reference gene *Caenorhabditis elegans* miR-39 (cel-39), a spike in control, was added to the serum sample and used as a technical quality control miR. Moreover 2 miR were selected for the hemolysis control [13]: miR-451a and miR-23a-3p. MiR-23a-3p is hemolysis independent since it is not expressed in the red blood cells (RBC) and therefore no change was anticipated independent on the hemolysis intensity. On the contrary, miR-451a is highly expressed in RBC and therefore the increase of this miR in serum is expected with the higher hemolysis levels. In order to define the level of hemolysis in each sample the ΔCt was calculated as presented in the formula below:

$\Delta Ct = Ct\ miR\text{-}23a\text{-}3p - Ct\ miR\text{-}451a$

Providing the samples showed no hemolysis $\Delta Ct \leq 5$, the range of $5 < \Delta Ct \leq 7$ indicated low risk of hemolysis and $\Delta Ct > 7$ designated high risk of hemolysis.

For the validation purpose 4 randomly selected donors were chosen. Each miR was measured in triplicate in 4 independent experiment setups.

The results obtained (supplementary figure 9) indicated that the most stable miR was hsa-miR-191-5p that confirmed the sequencing results. Moreover, we proved that the experiments were performed with reliable and stable technical standard if based on the cel-39 results. Therefore, for the final miR evaluation in serum miR-191 should be used as an internal control, cel-39 as a technical quality control miR and a combination of miR-451a and miR-23a-3p as a hemolysis control miRs.

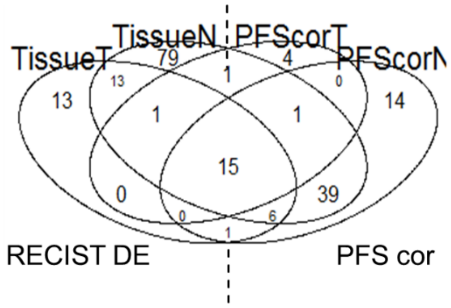

Supplementary Figure 1: Venn diagram summarizing RECIST differential expression analysis and PFS correlation analysis, each in tumor and normal tissue.

A

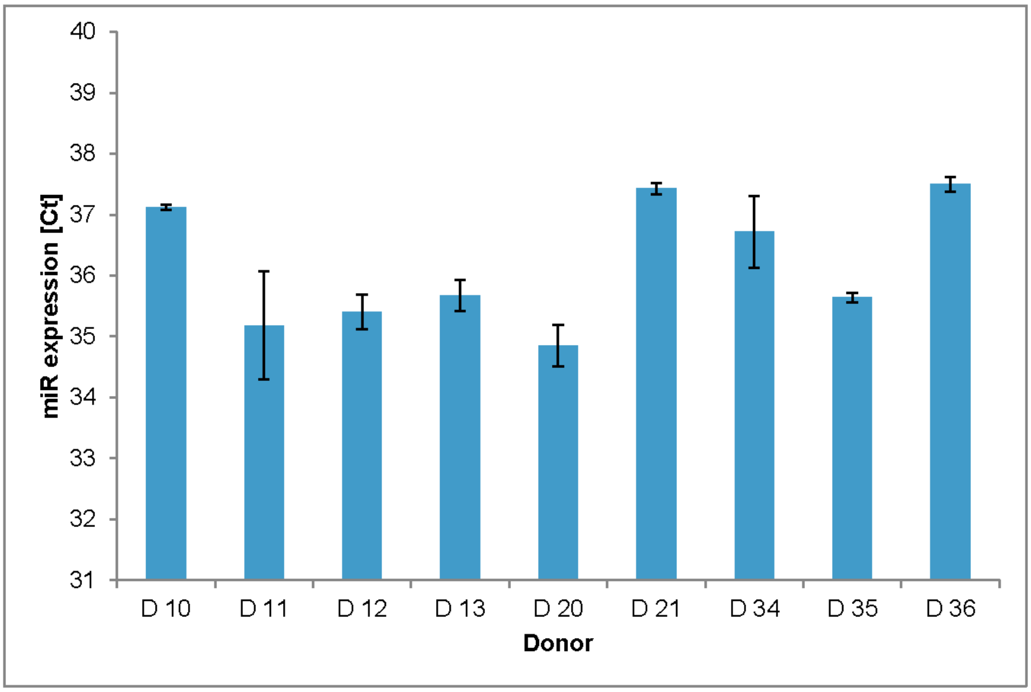

B

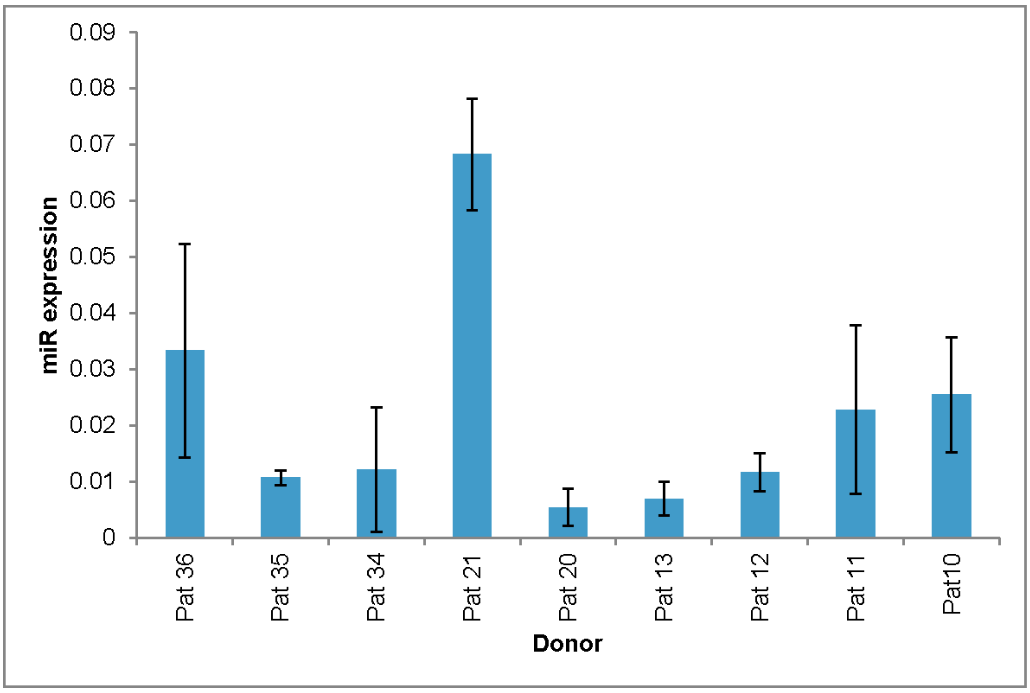

Supplementary Figure 2. RTqPCR results for the miR-99b-5p expression analysis in serum samples from 9 healthy donors. The high risk of hemolysis was noted only for D 20 (ratio of Ct miR-23a - Ct miR-451a >7). In the graph A Ct values are presented, graph B presents data normalized to miR-191 that proved to be the most stable reference miR among all tested.

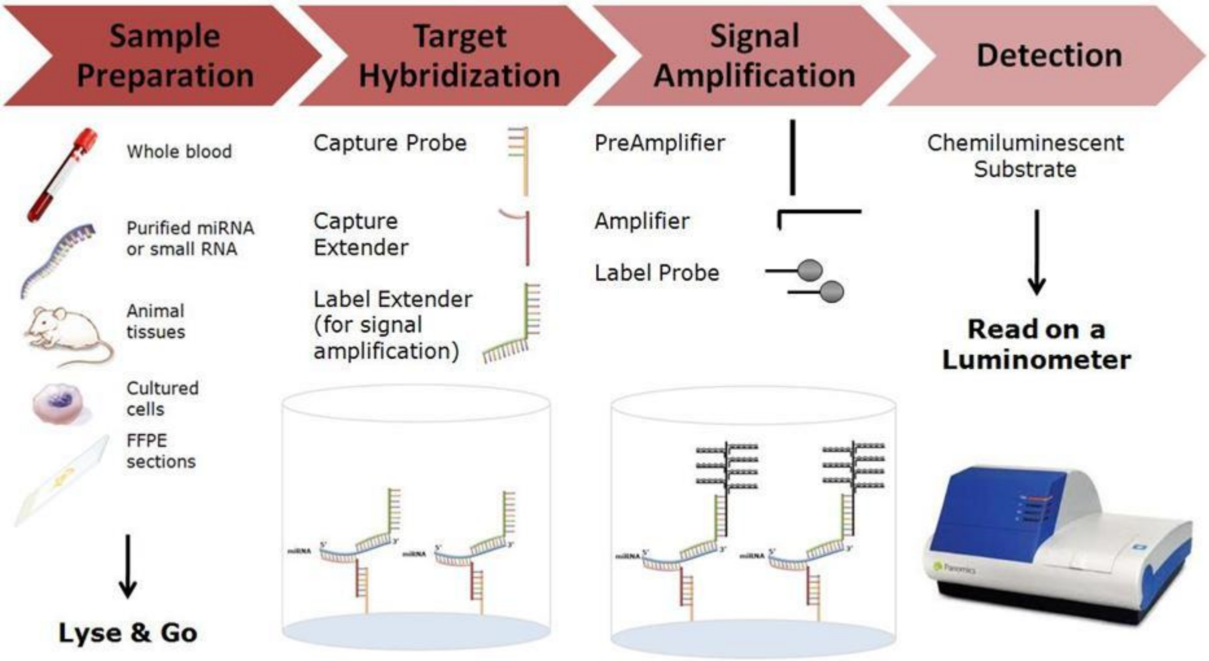

Supplementary Figure. 3. Affimetrix (QuantiGene® miRNA Assay) is a hybridization-based assay that quantifies miR targets. Initially, sample is lysed to release the RNAs and incubated with miR specific probe sets. As follows, the signal amplification tree is built via sequential hybridization of pre-amplifier, amplifier and label probe. Each amplification unit gives 400-fold signal amplification and there are six amplification units per target RNA copy leaning to 2400-fold signal amplification per copy RNA. The signal is detected by luminescence detector. Adapted from Affimetrix.

.

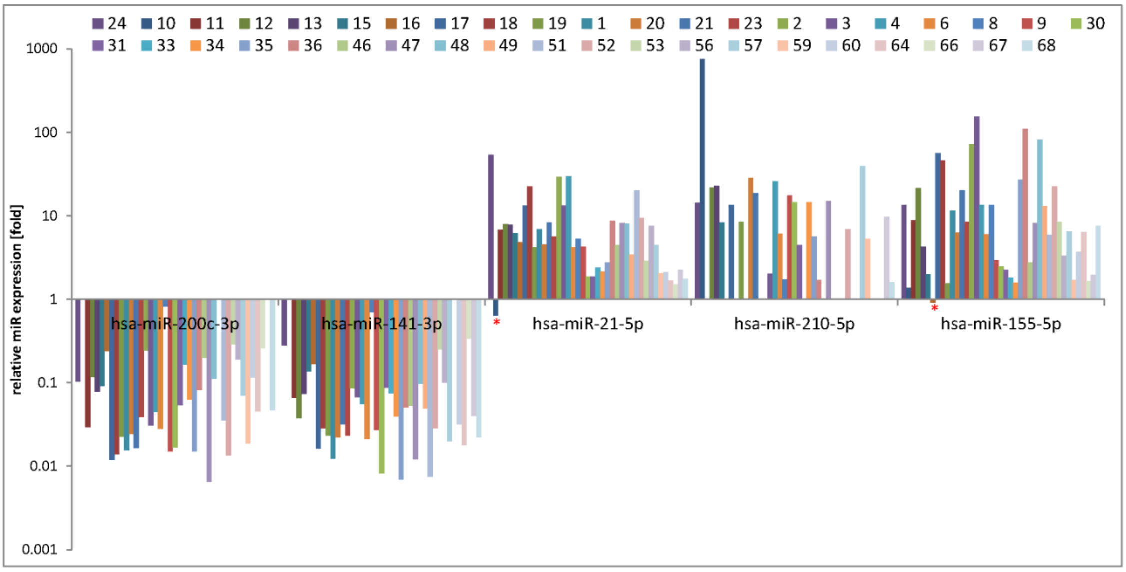

Supplementary Figure 4. MiRs' expression results obtained with sequencing platform. 5 control miRs selected based on the literature data [1-9] were analysed in 40 patients (Zürich cohort, n=20; Vienna cohort, n=20) selected for this step of the studies. MiR levels (Y axis) are expressed in the relative values of tumor vs. non-tumor adjacent tissue for the patient described in the legend. Value of 1 indicate no difference in tumor vs. non-tumor adjacent tissue, value >1 indicate up-regulation and <1 indicate a down-regulation of the miR expression in tumor tissue if compared to adjacent non-tumor tissue. Based on literature reports [1-9], expected deregulation of the miRs was down regulation and up regulation of miR-200c-3p, miR-141-3p and miR-21-5p, miR-210-5p, miR-155-5p respectively. For all patients the deregulation was as expected with two exceptions: Patient 10 and Patient 16 (indicated with *).

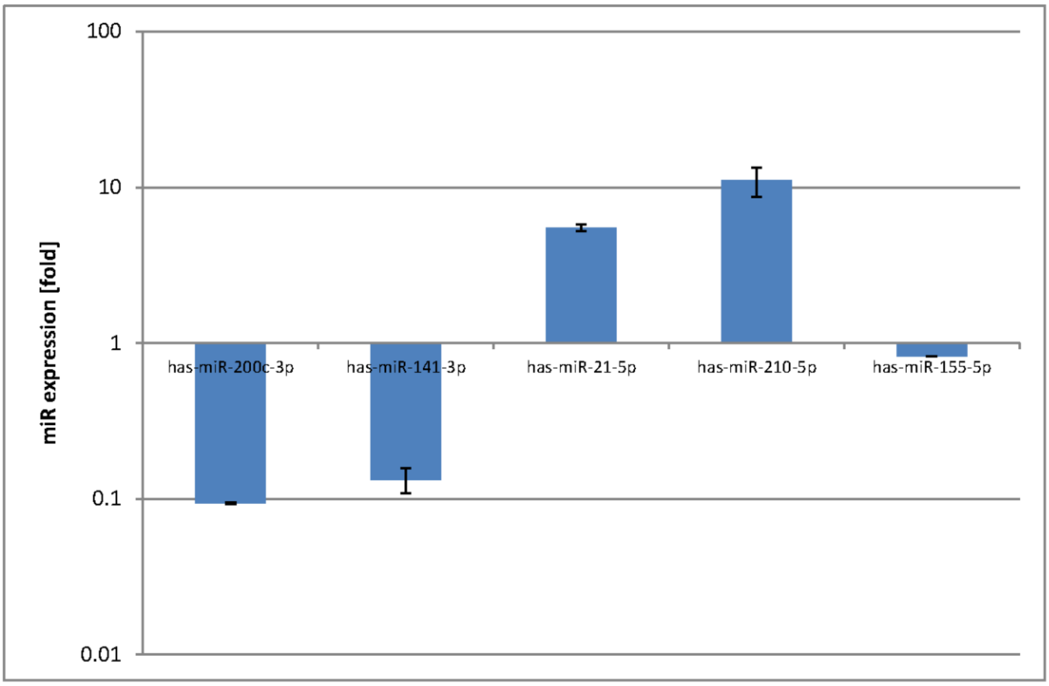

Supplementary Figure 5. Expression of the 5 control miRs selected based on the literature data [1-9] obtained with RTqPCR platform. MiR levels (Y axis) are expressed in the relative values of tumor vs. adjacent non-tumor tissue for the patient 16. Based on sequencing data down-regulation miR-200c-3p, miR-141-3p, miR-155-5p and up regulation of miR-21-5p, mir-210-5p was noted. For patient 16 the deregulation of all miRs confirmed the sequencing results. Error bars indicate the standard deviation obtained in 3 independent experiments.

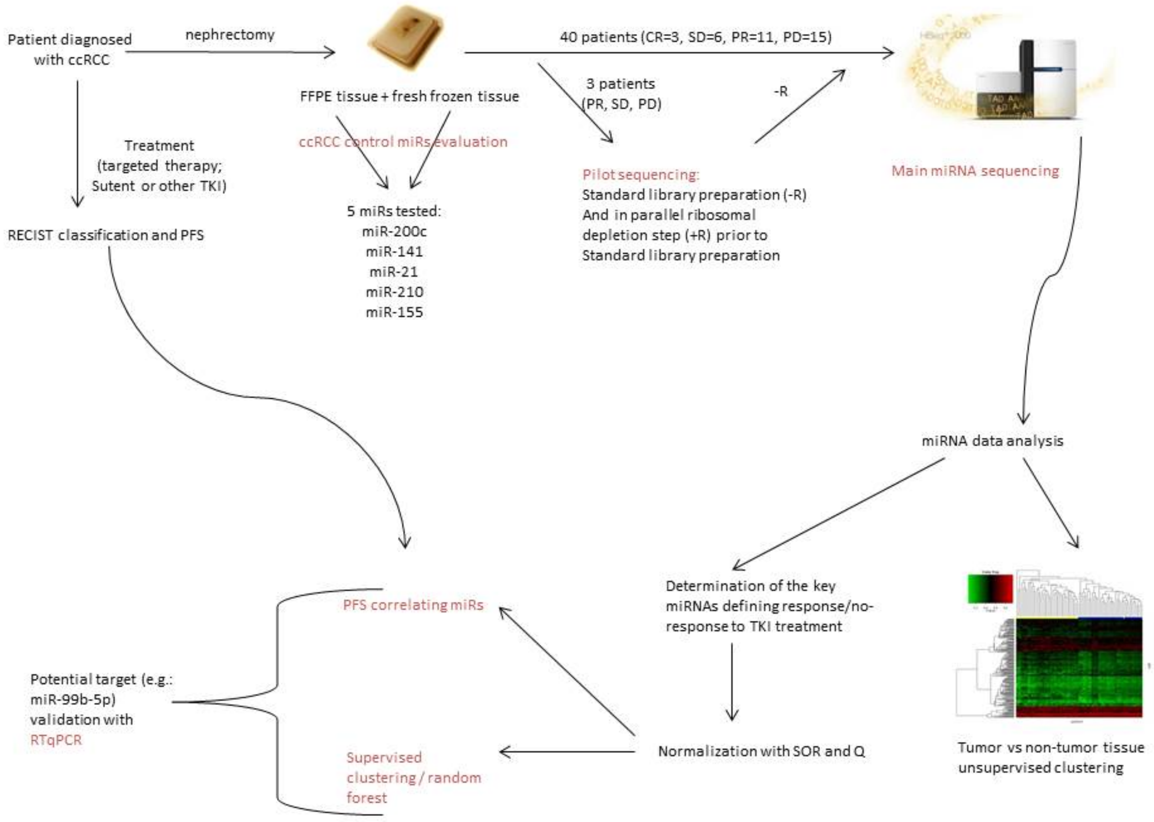

Supplementary Figure 6. Funnel figure presenting the workflow. Initially, patients diagnosed with renal tumor (later verified as ccRCC by pathological analysis) were submitted to surgery. Patients followed with the TKI treatment and were classified according to RECIST criteria as PD, SD, PR and CR.

I. The specimens obtained from the nephrectomy were fresh frozen and/or formalin fixed. The two types of tissue were compared in a ccRCC tissue testing in ccRCC control miRs evaluation stage.

II. a. For the main experimental phase, performed with miR sequencing platform 40 patients' FFPE tissues (tumor and non-tumor adjacent tissue from each patient) were selected.

b. Additionally, 3 patients were submitted to the sequencing pilot experiment, performed prior to the main sequencing study, where the ribosomal RNA depletion step was investigated in parallel to the standard library preparation protocol.

III. Data obtained as a result of global miR sequencing were analyzed using the

a. Unsupervised hierarchical clustering to analyze the separation of the tumor and non-tumor tissue.

b. MiR expression profiles were as well submitted to three variant normalization approaches: quintile and normalization via sum of reads and DES

eq2 normalization. As follows the results were correlated with the PFS data and

IIIc submitted to the supervised clustering/random forest analysis.

IV. The results for the top targets were validated with the RTqPCR platform.

Graphics partially adapted from [14]

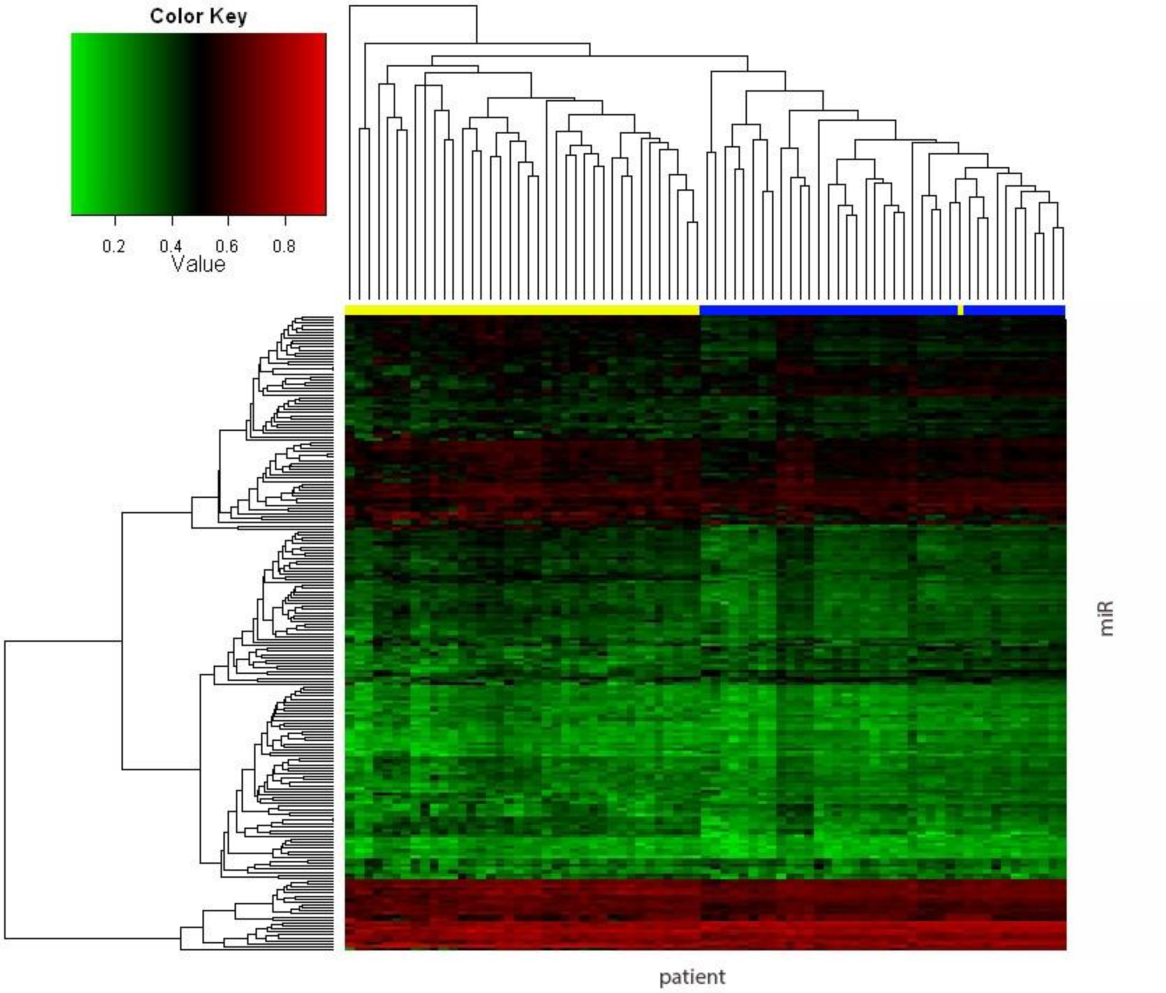

Supplementary Figure 7. Unsupervised hierarchical clustering of tumor (T – indicated yellow) and non-tumor (N - indicated blue) tissue of 40 patients based on the sequencing platform results. Patients were selected for this step based on the following criteria: i) availability of the PFS parameter (at the date of analysis); ii) RECIST classification to represent each RECIST group: PD, SD, PR, CR; iii) possibly highest number of patients with short and long PFS (the highest differences in miR expression were expected for the extreme phenotype groups). MiRs are presented in rows and patient samples in columns. The red and green colors provide information about up- or down-regulation, respectively. The intensity of the color in the heat map renders quantitative information about the change in expression level. The values are normalized to the miR expression (rows).

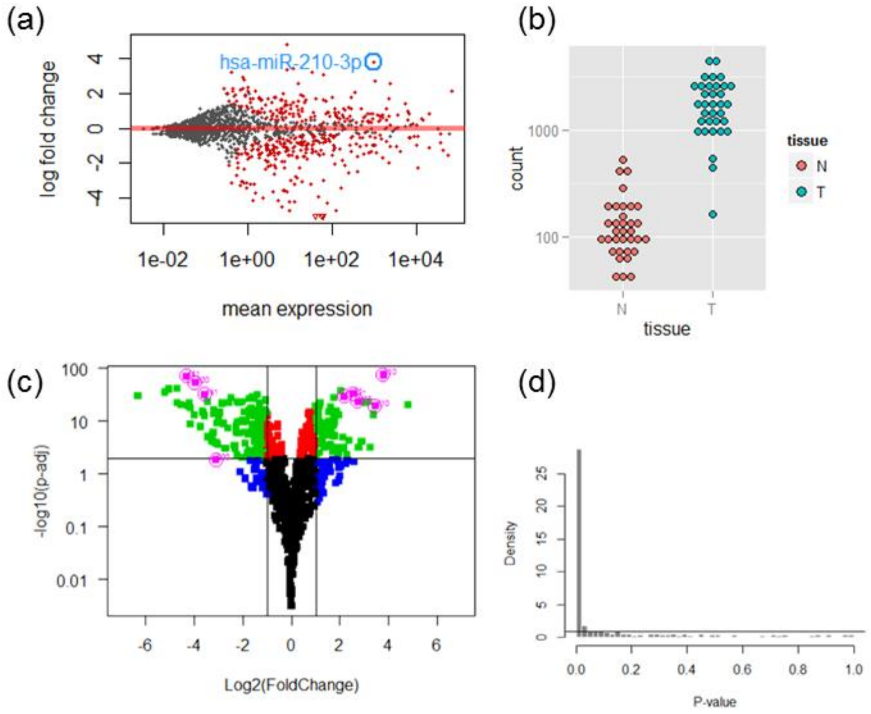

Supplementary Figure 8: Differential miR expression between normal and tumor tissue. (a) MvA plot with red dots indicating hits; miR-120-3p (blue) has the lowest p-value and its raw counts are displayed in (b). (c) Volcano plot showing significant (p<0.01) and strong (>2-fold) DE miRs (green), significant and weak (red), and insignificant >2-fold. (d) p-value histogram of the same data.

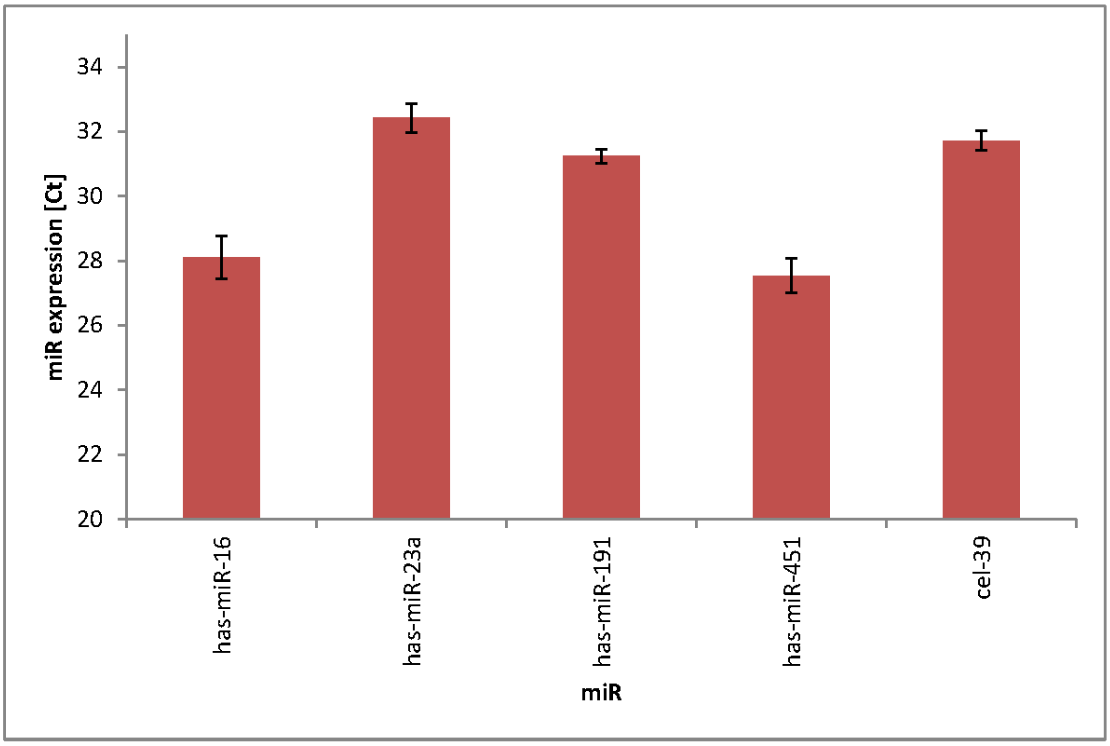

Supplementary Figure 9. Quantitative real time PCR results of 5 control miR candidates for serum analysis application. On the X axis candidate miRs are presented. On the Y axis miR expression is indicated (as Ct value). Error bars indicate the standard deviation measured for 4 different healthy donors analyzed in 4 independent experiments.

SUPPLEMENTARY TABLES

Supplementary Table 1. Results of normalization techniques (A: normalization via sum of reads - SOR B: quintile normalization – Q and C: DESeq2 normalization – Deseq2) applied to the sequencing results. In the presented table only miRs with >5 annotation score are presented (SOR and Q) and on average 1 read (DESeq2). In the top raw patient no., is indicated with an indication of tumor (T) or non-tumor (N) tissue; Second raw present the RECIST scoring: CR – complete response, PR – partial response, SD- stable disease, PD- progressive disease; values of the progression free survival (PFS) are presented in the third raw. First column list miRs that showed >5 reads for all analyzed patients (SOR and Q) or on average 1 read (DESeq2); na – the analysis of the patients' response according to the RECIST criteria is not feasible; (CR) – patients conditionally classified as CR

The table, due to its' size, is presented in attached excel file.

Supplementary table 2: Random Forest stability variable selection results. Column A sorted alphabetically, B sorted by decreasing importance.

A	B
hsa-miR-99a-5p-N	hsa-miR-324-3p-N
hsa-miR-99b-5p-T	hsa-miR-1271-5p-N
hsa-miR-99b-5p-N	hsa-miR-99b-5p-N
hsa-miR-100-5p-N	hsa-miR-100-5p-N
hsa-miR-100-5p-T	hsa-miR-409-5p-T
hsa-miR-145-3p-N	hsa-miR-145-3p-N
hsa-miR-199a-5p-N	hsa-miR-100-5p-T
hsa-miR-187-3p-T	hsa-miR-1296-5p-N
hsa-miR-324-3p-N	hsa-miR-501-3p-T
hsa-miR-328-3p-T	hsa-miR-199a-5p-N
hsa-miR-409-5p-N	hsa-miR-423-5p-N
hsa-miR-409-5p-T	hsa-miR-328-3p-T
hsa-miR-423-5p-N	hsa-miR-99b-5p-T
hsa-miR-501-3p-T	hsa-miR-99a-5p-N
hsa-miR-652-3p-N	hsa-miR-187-3p-T
hsa-miR-1271-5p-N	hsa-miR-409-5p-N
hsa-miR-1296-5p-N	hsa-miR-652-3p-N

Supplementary table 3: List of top 20 miRs (out of 98) with significant rank correlation ($p<0.05$) with PFS.

rank	miR	direction
1	hsa-miR-126-5p-N	up
2	hsa-miR-1268a-N	down
3	hsa-miR-1268b-N	down
4	hsa-miR-320a-N	down
5	hsa-miR-3613-5p-N	up
6	hsa-miR-615-3p-N	down
7	hsa-miR-99b-3p-N	down
8	hsa-miR-193b-3p-N	down
9	hsa-miR-222-3p-N	down
10	hsa-miR-99b-5p-T	up
11	hsa-miR-423-5p-N	down
12	hsa-miR-30c-2-3p-N	down
13	hsa-miR-328-3p-N	down
14	hsa-miR-374a-3p-N	up
15	hsa-miR-374a-5p-N	up
16	hsa-miR-501-3p-N	up
17	hsa-miR-320e-N	down
18	hsa-miR-148b-5p-T	down
19	hsa-miR-409-5p-N	up
20	hsa-miR-151a-3p-N	down

Supplementary table 4. Expression level of 5 control miRs in randomly selected 10 patients. Pearson correlation coefficient (R and R^2) of miR expression analyzed with RTqPCR (Mean value of three endogenous control RNAs (RNU44, RNU 48 and U6 snRNA) have been used as reference for normalization of miRs expression levels) and sequencing. bdl – indicate that the miR was below the test detection limit.

Patient	Target Name	RTqPCR	sequencing	R	R^2
46	miR 200c	0.077382	0.198404	0.997	0.995
	miR 141	0.062835	0.053523		
	miR 21	3.280549	4.499169		
	miR 210	22.37476	20.59351		
	miR 155	2.227664	2.74967		
53	miR 200c	0.582414	0.289094	0.992	0.985
	miR 141	0.36627	0.250898		
	miR 21	3.940031	2.893551		
	miR 210	2.494908	bdl		
	miR 155	8.393382	8.415064		
52	miR 200c	0.094798	0.013633	0.991	0.983
	miR 141	0.052266	0.028379		
	miR 21	14.22501	9.369851		
	miR 210	52.755	23.84659		
	miR 155	bdl	22.49608		
8	miR 200c	0.85799	0.815715	0.277	0.077
	miR 141	0.503377	0.708333		
	miR 21	bdl	5.29707		
	miR 210	294.0294	21.84615		
	miR 155	214732.6	13.49098		
19	miR 200c	0.059747	0.022499	0.994	0.989
	miR 141	0.030076	0.023157		
	miR 21	24.08961	4.227403		
	miR 210	95.67494	27.89828		

	miR 155	5.109095	1.556335		
20	miR 200c	0.115386	0.024385	0.971	0.942
	miR 141	0.083592	0.022121		
	miR 21	25.54584	4.526549		
	miR 210	87.12114	26.83925		
	miR 155	6.403239	6.337177		
2	miR 200c	0.004782	0.242397	0.999	0.998
	miR 141	bdl	0.086094		
	miR 21	4.459841	29.41222		
	miR 210	1.313131	6.007583		
	miR 155	10.26092	72.68638		
3	miR 200c	0.022706	0.030819	0.970	0.941
	miR 141	0.021344	0.066689		
	miR 21	2.384957	13.19345		
	miR 210	3.860757	11.25495		
	miR 155	12.42524	154.804		
4	miR 200c	0.104046	0.04476	0.896	0.802
	miR 141	0.083763	0.055385		
	miR 21	49.68001	29.78557		
	miR 210	75.71667	25.46229		
	miR 155	12.60753	13.53056		
12	miR 200c	0.011423	0.117792	0.137	0.019
	miR 141	0.007545	0.037557		
	miR 21	2.65337	8.016945		
	miR 210	8.065849	7.670647		
	miR 155	0.955296	21.57285		

Supplementary table 5. Patients' clinical data summary. PFS – progression free survival, OS- overall survival , SU - sunitinib, SO- Sorafenib, PZ- pazopanib, CR – complete response, PR – partial response, SD - stable disease, PD – progressive disease, TBD - to be determined, Y – yes, N – no, NA – not analyzable.

Patient	tumor type	age	age at diagnosis	sex (F- female, M- male)	stage	grade	PFS	Reason for treatment stop	OS	RECIST	therapy	serum	sequencing
**USZ cohort**													
1	clear cell	60	55	M	pT2b	3	13.16	progression	14.8	PD	PZ	Y	Y
2	clear cell	alive	38	F	pT1a	3	2.07	progression	alive	PD	PZ	N	Y
3	papillary type II	58	57	M	pT1b	3	4.39	progression	6.9	PD	SU	N	Y
4	clear cell	66	64	M	pT3a	3	7.20	progression	26.1	PD	PZ	N	Y
5	clear cell	59	46	M	pT2	2	17.46	progression	41.3	PD	SO	N	N
6	clear cell	alive	63	F	pT3a	3	16.69	progression	alive	PD	PZ	N	Y
8	clear cell /sarkomatoid	56	54	M	pT3a	4	7.16	progression	23.0	PD	SO	Y	Y
9	clear cell	68	64	M	pT3a	4	12.43	progression	55.7	PD	PZ	Y	Y
10	clear cell	alive	58	M	pT3a	3	NA	progression	alive	PR	PZ	N	Y
11	clear cell	75	71	M	pT4	3	9.52	progression	53.1	PD	SU	N	Y
12	clear cell	76	63	M	pT3b	4	7.46	progression	18.7	PD	SU	N	Y
13	clear cell	75	70	M	pT3a	1	7.36	progression	64.3	PD	SO	N	Y
15	papillary type II	54	51	M	pT3c	4	6.20	progression	34.5	PD	SU	N	Y
16	clear cell	66	65	M	pT3b	3	7.87	progression	13.9	PD	SU	N	Y

17	clear cell with pappillary	60	58	F	pT2	3	NA	CR reported	22.4	CR	SU	N	Y
18	clear cell	57	54	M	pT1a	4	NA	progression	36.5	NA	SU	N	Y
19	clear cell	37	35	F	pT3a	3	9.49	progression	19.9	PD	PZ	N	Y
20	clear cell	alive	62	M	pT3a	3	14.66	progression	alive	PD	SU	N	Y
21	clear cell	58	51	M	pT2	3	38.10	progression	88.7	PD	SU	N	Y
22	clear cell	72	68	M	pT3b	3	7.93	progression	48.2	PD	SU	N	N
23	clear cell	73	62	F	pT2	2	NA	progression	23.8	NA	SU	N	Y
24	papillary type II	76	75	F	pT3a	3	4.16	progression	14.5	PD	SO	N	Y
**Vienna cohort**													
30	clear cell/sarkomatoid	na	na	M	pT3a	2	17.5	progression	20.6	PR	SU	N	Y
31	clear cell	na	na	F	pT1	2	10.0	progression	17.9	SD	SU	N	Y
32	clear cell	na	na	M	pT1a	2	14.0	progression	39.8	SD	SU	N	N
33	clear cell	na	na	M	pT1b	3	19.7	progression	33.3	SD	SU	N	Y
34	clear cell	na	na	M	pT3b	3	16.7	progression	16.7	PR	SU	N	Y
35	clear cell	na	na	M	pT3b	3	8.4	progression	10.6	PR	SU	N	Y
36	clear cell	na	na	M	pT3b	4	23.7	progression	38.6	PR	SU	N	Y
37	clear cell	na	na	M	pT3a	2	3.9	progression	6.3	PD	SU	N	N
38	clear cell	na	na	F	pT3a	2	NA	progression	37.7	SD	SU	N	N
39	clear cell	na	na	M	pT3a	3	32.2	progression	32.2	PR	SU	N	N
40	clear cell	na	na	M	pT4	4	4.5	progression	4.5	PD	SU	N	N
41	clear cell	na	na	M	pT3b	3	3.0	progression	18.4	PD	SU	N	N
42	sarkomatoid	na	na	M	pT3a	4	3.0	progression	5.5	PD	SU	N	N
43	sarkomatoid	na	na	F	pT3a	4	7.0	progression	7.0	SD	SU	N	N
45	clear cell	na	na	F	pT3a	3	17.5	progression	40.9	SD	SO	N	N
46	clear cell	na	na	M	pT3a	2	45.3	progression	45.3	PR	SU	N	Y
47	clear cell	na	na	M	pT3b	3	NA	progression	49.8	PR	SU	N	Y
48	clear cell	na	na	F	pT3b	3	48.0	CR reported	48.0	CR	SU	N	Y

49	clear cell	na	na	M	pT3b	3	14.9	progression	38.4	SD	SU	N	Y
50	clear cell	na	na	F	pT3b	3	2.8	progression	10.8	PD	SU	N	N
51	clear cell	na	na	M	pT3a	3	20.6	progression	20.6	PR	SU	N	Y
52	clear cell	na	na	F	pT3a	3	11.3	progression	14.0	SD	SU	N	Y
53	clear cell	na	na	M	pT3a	2	26.2	CR reported	NA	CR	SU	N	Y
54	clear cell/eosiNphile granular	na	na	F	pT3b	3	28.0	progression	57.5	PR	SO	N	N
55	clear cell	na	na	F	pT4	4	12.2	progression	26.9	PR	SO	N	N
56	clear cell	na	na	F	pT2	2	66.1	progression	66.1	PR	SU	N	Y
57	clear cell	na	na	M	pT3a	2	27.0	progression	27.0	SD	SU	N	Y
58	clear cell	na	na	M	pT3b	2	5.4	progression	57.9	PD	SU	N	N
59	clear cell	na	na	M	pT1	2	18.6	progression	22.2	PR	SU	N	Y
60	clear cell	na	na	M	pT3b	2	34.2	progression	34.6	PR	SU	N	Y
61	clear cell	na	na	F	pT3a	3	1.4	progression	1.4	PD	SU	N	N
62	clear cell	na	na	M	pT3b	2	7.5	progression	20.5	SD	SU	N	N
63	clear cell	na	na	F	pT3a	3	7.4	progression	7.4	SD	SU	N	N
64	clear cell	na	na	M	pT3a	3	47.9	CR reported	47.9	CR	SU	N	Y
65	clear cell	na	na	M	pT3a	3	1.9	progression	10.6	PD	SU	N	N
66	clear cell	na	na	F	pT1a	1	27.5	progression	56.4	PR	SU	N	Y
67	clear cell	na	na	F	pT3b	3	47.4	progression	77.0	SD	SU	N	Y
68	clear cell	na	na	F	pT3a	3	11.3	progression	27.7	PR	SU	N	Y
69	clear cell	na	na	M	pT3b	3	20.1	progression	34.4	PR	SU	N	N
**Tuebingen cohort**													
70	clear cell	alive	71	M	pT3a	3	0.69	side effects	NA	NA	SU	Y	N
71	clear cell	52	51	M	pT1a	3	1.05	progression	4.4	PD	SU	Y	N
72	clear cell	49	49	F	pT3b	3	1.25	progression	1.9	PD	SU	N	N
73	clear cell	56	56	M	pT4	3	1.64	progression	3.1	PD	SU	N	N
74	clear cell	54	53	F	pT4	3	2.85	progression	7.1	PD	SU	Y	N

75	clear cell	alive	57	M	pT3a	2	3.15	side effects	NA	PR	SU	N	N
76	clear cell	74	73	M	pT1a	2	4.43	side effects	10.8	PD	SU	Y	N
77	clear cell	68	66	M	pT3a	3	5.15	side effects	34.7	PD	SU	Y	N
78	clear cell	57	56	M	pT3b	3	5.74	progression	13.8	PR	SU	Y	N
79	clear cell	alive	71	F	pT1b	2	2.79	progression	NA	PD	SU	Y	N
80	clear cell	52	50	M	pT3a	3	7.25	progression	18.1	PR	SU	N	N
81	clear cell	alive	86	F	pT3a	2	7.77	progression	NA	PD	SU	Y	N
82	clear cell	alive	52	M	pT3a	3	9.02	progression	NA	SD	SU	N	N
83	clear cell	57	49	M	pT1b	2	9.05	progression	23.1	SD	SU	N	N
84	clear cell	75	73	M	pT2b	2	9.77	progression	17.4	PR	SU	N	N
85	clear cell	72	70	M	pT3b	2	9.84	progression	24.4	SD	SU	Y	N
86	clear cell	69	66	M	pT3b	2	9.90	progression	31.2	PR	SU	Y	N
87	clear cell	67	65	F	pT3b	3	11.11	progression	14.2	PR	SU	N	N
88	clear cell	alive	47	M	pT2b	3	11.15	progression	NA	PR	SU	Y	N
89	clear cell	84	79	M	pT3b	2	11.38	progression	48.5	CR	SU	N	N
90	clear cell	49	47	M	pT3a	2	12.23	progression	20.6	PR	SU	Y	N
91	clear cell	alive	56	F	pT1b	3	36.39	ongoing treatment/PFS censored	NA	PR	SU	Y	N
92	clear cell	alive	70	M	pT3a	3	14.69	progression	NA	PR	SU	Y	N
93	clear cell	alive	68	M	pT3b	3	19.51	CR reported	NA	CR	SU	N	N
94	clear cell	alive	71	M	pT1b	2	27.05	progression	NA	SD	SU	Y	N
95	clear cell	alive	61	F	pT3b	2	27.08	progression	NA	PR	SU	N	N
96	clear cell	alive	52	F	pT1b	2	44.89	progression	NA	PR	SU	N	N
97	clear cell	alive	72	M	pT3a	3	60.10	CR reported	NA	CR	SU	Y	N
98	clear cell	alive	66	M	pT1	1	2.07	progression	NA	PD	SU	N	N

Supplementary table 6. Results obtained by the computation analysis of the raw sequencing data are presented in the table. N - Indicate the non-tumor tissue from the annotated patient sample, T - indicate the tumor tissue from the annotated patient sample. First column list miRs, second present miRBase accession number (MIMAT number)

The table, due to its' size, is presented in attached excel file.

Supplementary table 7. Relative expression of miR-21, miR-210, miR-155, miR-141 and miR-200c in FFPE and fresh frozen tumor tissue analyzed with RTqPCR and hybridization based assay. Presented values are expressed as fold change of miR in tumor vs. adjacent non-tumor tissue. FFPE – formalin fix paraffin embedded.

	RTqPCR		Hybridization based assay	
	Fresh frozen	FFPE	Fresh frozen	FFPE
miR-21	1.2	2.7	5.0	5.1
miR-210	5.0	14.2	102235	140752.5
miR-155	2.4	22.2	29392	42590.5
miR-200c	0.04	0.03	0.00017	0.00008
miR-141	0.01	0.03	0.00030	0.00009

SUPPLEMENTARY REFERENCES

1. Catto JW, Alcaraz A, Bjartell AS, De Vere White R, Evans CP, Fussel S, Hamdy FC, Kallioniemi O, Mengual L, Schlomm T, Visakorpi T. MicroRNA in prostate, bladder, and kidney cancer: a systematic review. *Eur Urol* 2011;**59**: 671-81.

2. Juan D, Alexe G, Antes T, Liu H, Madabhushi A, Delisi C, Ganesan S, Bhanot G, Liou LS. Identification of a microRNA panel for clear-cell kidney cancer. *Urology* 2010;**75**: 835-41.

3. Jung M, Mollenkopf HJ, Grimm C, Wagner I, Albrecht M, Waller T, Pilarsky C, Johannsen M, Stephan C, Lehrach H, Nietfeld W, Rudel T, et al. MicroRNA profiling of clear cell renal cell cancer identifies a robust signature to define renal malignancy. *J Cell Mol Med* 2009;**13**: 3918-28.

4. Liu H, Brannon AR, Reddy AR, Alexe G, Seiler MW, Arreola A, Oza JH, Yao M, Juan D, Liou LS, Ganesan S, Levine AJ, et al. Identifying mRNA targets of microRNA dysregulated in cancer: with application to clear cell Renal Cell Carcinoma. *BMC Syst Biol* 2010;**4**: 51.

5. Nakada C, Matsuura K, Tsukamoto Y, Tanigawa M, Yoshimoto T, Narimatsu T, Nguyen LT, Hijiya N, Uchida T, Sato F, Mimata H, Seto M, et al. Genome-wide microRNA expression profiling in renal cell carcinoma: significant down-regulation of miR-141 and miR-200c. *J Pathol* 2008;**216**: 418-27.

6. Weng L, Wu X, Gao H, Mu B, Li X, Wang JH, Guo C, Jin JM, Chen Z, Covarrubias M, Yuan YC, Weiss LM, et al. MicroRNA profiling of clear cell renal cell carcinoma by whole-genome small RNA deep sequencing of paired frozen and formalin-fixed, paraffin-embedded tissue specimens. *J Pathol* 2010;**222**: 41-51.

7. White NM, Bao TT, Grigull J, Youssef YM, Girgis A, Diamandis M, Fatoohi E, Metias M, Honey RJ, Stewart R, Pace KT, Bjarnason GA, et al. miRNA profiling for clear cell renal cell carcinoma: biomarker discovery and identification of potential controls and consequences of miRNA dysregulation. *J Urol* 2011;**186**: 1077-83.

8. Neal CS, Michael MZ, Rawlings LH, Van der Hoek MB, Gleadle JM. The VHL-dependent regulation of microRNAs in renal cancer. *BMC Med* 2010;**8**: 64.

9. Valera VA, Walter BA, Linehan WM, Merino MJ. Regulatory Effects of microRNA-92 (miR-92) on VHL Gene Expression and the Hypoxic Activation of miR-210 in Clear Cell Renal Cell Carcinoma. *J Cancer* 2011;**2**: 515-26.

10. TruSeq small RNA sample preparation guide. http://support.illumina.com/content/dam/illumina-support/documents/documentation/chemistry_documentation/samplepreps_truseq/truseqsmallrna/truseq-small-rna-sample-prep-guide-15004197-f.pdf, vol. 2015: ILLUMINA PROPRIETARY.

11. Ribo-Zero rRNA Removal Kit (Human/Mouse/Rat) http://www.epibio.com/docs/default-source/protocols/ribo-zero-rrna-removal-kit-%28human-mouse-rat%29.pdf?sfvrsn=6, vol. 2015.

12. Wu X, Weng L, Li X, Guo C, Pal SK, Jin JM, Li Y, Nelson RA, Mu B, Onami SH, Wu JJ, Ruel NH, et al. Identification of a 4-microRNA signature for clear cell renal cell carcinoma metastasis and prognosis. *PloS one* 2012;**7**: e35661.

13. Profiling on microRNA in serum/plasma and other biofluids www.exiqon.com.

14. Illumina http://www.illumina.com/science/education/adventures-in-genomics.html.
